# Prevalence of depression in melasma: a systematic review and meta-analysis

**DOI:** 10.3389/fpsyt.2023.1276906

**Published:** 2024-01-08

**Authors:** Wenjing Chen, Yue Wan, Yuan Sun, Changyong Gao, Jianhong Li

**Affiliations:** ^1^First Clinical Medical College of Beijing University of Chinese Medicine, Beijing, China; ^2^Department of Dermatology, Dongzhimen Hospital, Beijing University of Chinese Medicine, Beijing, China

**Keywords:** melasma, depression, prevalence, systematic review, meta-analysis

## Abstract

**Background:**

Due to cosmetic disfigurement, melasma can negatively affect the quality of life and emotional and mental health, further leading to depression.

**Objective:**

Prevalence rates of depression in patients with melasma vary widely across studies. The aim of this systematic review and meta-analysis was to estimate the prevalence of depression among melasma patients.

**Methods:**

The PubMed, Embase, Web of Science, and Scopus databases were searched to identify articles evaluating the prevalence of depression in melasma patients from their inception to 12 July 2023. Studies were reviewed in accordance with the Preferred Reporting Items for Systematic Reviews and Meta-Analyses (PRISMA) guidelines, and a meta-analysis was performed using the Stata 14.0 software.

**Results:**

Sixteen studies met the eligibility criteria out of the 859 studies, containing a total of 2,963 melasma patients for this systematic review and meta-analysis. Meta-analyses revealed that the pooled prevalence of depression among patients with melasma was 43.4% (95% CI 30.5–56.2%, *Q*-value = 808.859, d.f. = 15, *p* < 0.001, tau^2^ = 0.065, *I^2^* = 98.1%). The meta-regression found that the publication year, sample size, and study quality were not significant moderators for the observed heterogeneity in prevalence. A subgroup analysis according to depression assessment methods showed that the prevalence of depressive disorders was 24.2% (95% CI 16.8–31.6%), and the prevalence of depressive symptoms was 45.1% (95% CI 31.2–59.0%). A subgroup analysis by geographic regions showed that patients in Asia had the highest prevalence of depression at 48.5% (95% CI 26.0–71.0%), compared to other regions. A subgroup analysis by study design showed that the prevalence of depression in case–control studies was almost identical to cross-sectional studies. In the case of OR, the pooled OR of depression between patients with melasma and health controls was 1.677 (95% CI 1.163–2.420, *p* = 0.606, *I^2^* = 0.0%).

**Conclusion:**

The prevalence of depression was relatively high in patients with melasma, and there was a correlation between melasma and depression, encouraging clinicians to screen for depression in their patients and providing a combination of physical and psychosocial support. If necessary, they should be referred to formal mental health services to seek professional psychological intervention.

**Systematic review registration:**

https://www.crd.york.ac.uk/PROSPERO/, CRD42022381378.

## 1 Introduction

Melasma, also known as chloasma or mask of pregnancy, is a common chronic acquired hyperpigmentation disorder that predominantly affects women of childbearing age (1). It most often presents as symmetric irregular light-to-dark brown macules and patches that occur primarily on sun-exposed areas of the face. It is more prevalent in Fitzpatrick skin types III–V [Fitzpatrick skin type is a numerical classification used for measuring human skin pigmentation and sun sensitivity (2)], thus more often seen in Asians and Latin Americans (3). Although the precise cause of melasma remains elusive, however, multiple factors can trigger or aggravate it: ultraviolet radiation exposure, endocrine factors, genetic factors, medications containing phototoxic agents, ingredients of certain cosmetics, and psychological stress (4). Histologically, increased pigment may be situated epidermally, dermally, or on both sites. In the epidermal type, there is an increase of melanin throughout the layers of the epidermis, particularly in the basal and supra-basilar layers. Melanocytes are generally enlarged, increased in activity, and have distinct dendrites. Meanwhile, there is an increase in melanosomes and an acceleration in the movement of melanin toward keratinocytes. In the dermal type, melanin enters the papillary dermis and is taken up by macrophages (5). Additionally, it also involves changes in keratinocytes, fibroblasts, endothelial cells, and the basement membrane (3). The diagnosis of melasma is generally clinical. There is no specific therapy that is universally effective for patients with melasma. The objective of melasma management is to prevent further hyperpigmentation, reduce melanin production, and accelerate melanin excretion (6).

Melasma is a common disfiguring cutaneous disorder, as its facial occurrence and easy visibility can cause an aesthetic impact on the patient’s appearance, which can easily lead to depression in patients with melasma. Depression is a common psychological issue and a negative emotional state that can erode patients’ sense of happiness and lower their quality of life. It can lead to the deterioration of skin disease, and the resulting burden of worsening skin disease can exacerbate depression (7). Research has shown that persistent psychological stress due to melasma often leads to depression (8) or that melanin production is affected as part of the local response of the same hypothalamic–pituitary axis to stress as depression (9). Additionally, research has pointed out that depression can raise the levels of cortisol and pro-opiomelanocortin, which have melanogenic potential and cause an increase in melanin content in melanocytes (10). Despite the presence of multiple studies examining the prevalence of depression among these patients, there is no meta-analysis of the prevalence of depression in melasma and only one meta-analysis on the quality of life between them (11). This meta-analysis, therefore, aims to estimate the pooled prevalence of depression among patients with melasma that will hopefully raise the awareness of clinicians, facilitate screening of patients, and provide referrals for treatment when necessary.

## 2 Methods

### 2.1 Protocol and registration

The project was registered as a protocol with PROSPERO (CRD42022381378) and adhered to the PRISMA reporting guidelines (12). The study was exempt from review and approval by an ethics committee because the data were obtained from publicly available databases.

### 2.2 Search strategy

The PubMed, Embase, Scopus, and Web of Science databases were systematically searched from inception to 12 July 2023 to identify all relevant studies on the prevalence of depression in melasma patients, regardless of any restrictions on publication date and language. The search strategy combined outcomes of interest (depression) and population of interest (melasma patients). The specific search terms were summarized as follows: “Depression,” “Depressive Disorder,” “Depressive Disorder, Major,” “Dysthymic Disorder,” “Depressive Symptom,” “Bipolar Disorder,” “Mood Disorders,” “Mental Health,” “Antidepressive Agents,” “Adjustment Disorders,” “Psychological Distress,” “Emotional Depression,” “Melasma,” “Chloasma,” “Melanosis,” “Melanism,” and “Freckle.” The retrieval steps for PubMed are shown in Supplementary material 1. In addition, reference lists of relevant studies and reviews were screened manually to identify potentially omitted articles.

### 2.3 Study selection

Studies were eligible for inclusion if they fulfilled the following criteria according to the participants, intervention, comparison, outcomes, and study design (PICOS) guidelines: participants (P): the study involved patients with melasma; intervention (I): not applicable; comparison (C): not applicable; outcomes (O): the study reported data for depression as defined by validated self-reported scales, diagnostic interviews by a psychiatrist based on I.C.D-10 (International Classification of Diseases by W.H.O 1992), or self-reported medical history (taking antidepressants); and study design (S): the study was an observational study, such as a cross-sectional, cohort, or case–control study. Additionally, the following exclusion criteria were applied: (1) The study did not report the prevalence of depression among the sampled patients with melasma. (2) The data were duplicated, incomplete, or irrelevant.

### 2.4 Data extraction

All of the records identified through the electronic database search were downloaded and imported into the EndNote literature management software. First, duplicate studies were eliminated. Then, the titles and abstracts were independently read for preliminary screening by two independent researchers. Finally, they reviewed the full text in the remaining studies to confirm compliance with the eligibility criteria for inclusion and exclusion and established an Excel spreadsheet for data extraction, which was independently completed by four researchers. The extracted data were as follows: (1) publication details (title; author; and year); (2) details of the included patients (age; region; gender; setting; percentage of marriage or cohabitation; sample size; and positive patients); (3) details of the criteria used for defining depression cases (self-reported scales; diagnostic interviews; and medical history); and (4) other details (study design; relevant subgroup data; and type of control group). For some studies lacking data, we attempted to contact the study authors to obtain more information. However, we have only received a response from one study author stating that the study author was unable to provide raw data as this was a preliminary study. We have not received a response from another research author. After mutual consultation among our research team members, these studies were excluded.

### 2.5 Quality assessment

Study quality was assessed using Joanna Briggs Institute (JBI) Critical Appraisal Checklist for Studies Reporting Prevalence Data (13). The tool contains nine items and covers essential domains of population, measurement, and statistical approach. Each item was scored with “Yes” (1), “No” (0), and “Unclear” (indicating no report information) (0), and the quality score range was 0 to 9 points. Studies were classified as high quality (total score > 5), medium quality (total score 4 or 5), and low quality (total score < 4) (14). For the third item in the list (“Was the sample size adequate?”), the formula recommended in prevalence studies was used to calculate the minimum sample size ( $n=Z^{2}\times P\left(1-P\right)÷d^{2}$ ), where n meant the sample size, Z was the statistic corresponding to the level of confidence and was assigned 1.96, P represented expected prevalence, which was specified as 0.4, d meant precision, and the allocation was 0.05 (15). Based on the above formula, a minimum sample size of 369 participants was required. Each study was evaluated by two independent authors, and disagreements were resolved by a third reviewer.

### 2.6 Statistical analysis

The pooled prevalence, odds ratio (OR), and corresponding 95% confidence interval (CI) were calculated; and the forest plots were produced to visualize the results. Q-statistic and I^2^-statistic were used to test for heterogeneity across studies. The fixed-effect model was chosen to calculate pooled analyses if *p* ≥ 0.10 and *I^2^* ≤ 50%, indicating low heterogeneity, whereas a random-effect model was applied in case of significant heterogeneity (*p* < 0.10 and *I^2^* > 50%). A subgroup analysis was undertaken to explore potential sources of heterogeneity according to depression assessment methods, geographic regions, and study design only if there were at least two studies in each subgroup category. A meta-regression analysis was performed to explore potential moderators that might explain between-study heterogeneity. The covariates in the meta-regressions included the publication year, the sample size, and the study quality. Potential publication bias was assessed with Egger’s test and funnel plots for visual inspection. Sensitivity analysis to test the influence of a single study on the overall pooled estimate, by excluding each study step by step, was used to evaluate the robustness of the overall pooled estimate. A two-sided test and a significant level of 0.05 were used. All statistical analyses were done using Stata 14.0 statistical software.

## 3 Results

### 3.1 Selection of studies

**Figure 1:**
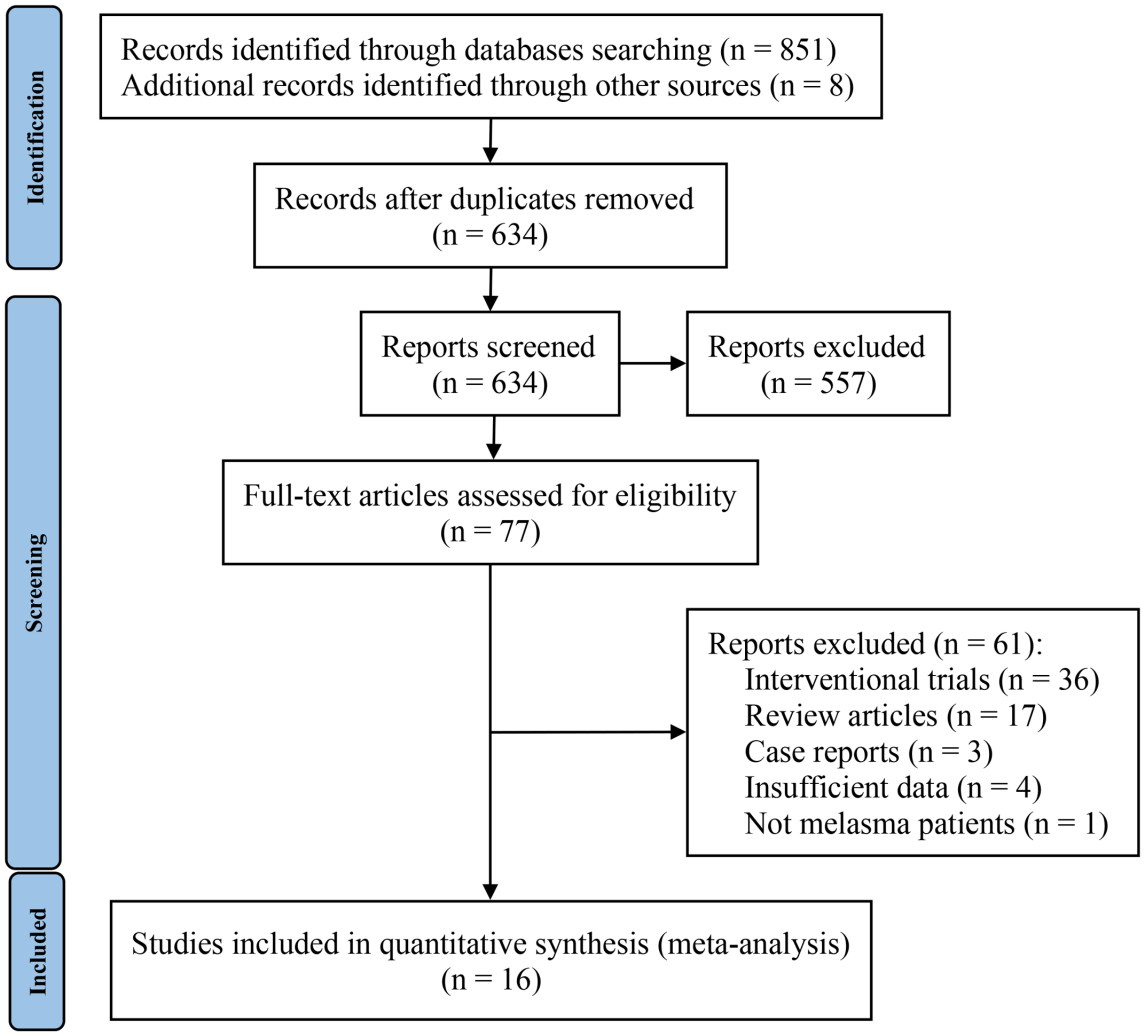
PRISMA flow diagram of studies’ screening and selection.

The screening process is shown in the PRISMA flowchart in Figure 1. A total of 859 studies were identified, 851 of which were identified through database searches and the remaining 8 studies were from reference lists. After identifying 225 duplicate records, all remaining studies were carefully screened through their titles and abstracts; 557 studies were excluded during this step. A total of 77 studies were given a full-text review to determine eligibility; those omitted to satisfy inclusion and exclusion criteria were excluded. Finally, 16 studies were included in the meta-analysis. Interrater reliability between reviewers for study selection was high (Kappa = 0.725, *p* < 0.01).

### 3.2 Characteristics of included studies

The general characteristics of the included studies are shown in Table 1. A total of 16 studies were included, containing a total of 2,963 individuals with melasma. Included studies were published between 2010 and 2023, along with 13 articles, 2 abstracts (27, 29), and 1 letter (19). There were two study designs, namely, cross-sectional studies and case–control studies. The mean age of melasma patients was reported in 12 studies ranging from 31.29 (21) to 48.33 years (16); the remaining 4 studies indicated that participants were all adults (17, 20, 30, 31). Thirteen studies reported the sex of melasma participants, of which five were all female patients (19, 22, 23, 27, 29), and eight had a diverse female proportion ranging from 67.44 (20) to 97.07% (31). One study was population-based (19), while the rest of the studies were hospital-based. Among 16 studies, 10 studies were conducted in Asia, 4 in the Americas (19, 22, 26, 29), and 2 in others [Europe (16) and Africa (27)]. To assess depression, eight studies used the Hospital Anxiety and Depression Scale (HADS) (16, 17, 19, 21, 22, 24, 27, 29), three used the Patient Health Questionnaire-9 (PHQ-9) (18, 20, 25), two used the Self-rating Depression Scale (SDS) (23, 31), one used the Hamilton Depression Scale (HDRS) (28), one used the International Classification of Diseases-WHO 1992 (I.C.D-10) (30), and one was based on medical history (taking antidepressants) (26). Four studies reported the prevalence of depression in healthy controls (16, 22, 24, 26). When evaluated by JBI quality assessment criteria, seven studies were rated as high quality (16, 18, 21–24, 28), six were rated medium quality (19, 20, 25–27, 31), and three were rated low quality (17, 29, 30). The details of the quality assessment for the 16 included studies are presented in the Supplementary Table 1.

**Table 1:** Characteristics of included studies.

Author	Region	Design	Sample size	Age (year)	Female	Prevalence (%)	Tool
Platsidaki 2023 (16)	Europe	Case–control	127	48.33 ± 18.39	121	34.7%	HADS
Kumar 2023 (17)	Asia	Case–control	30	NR	NR	70.0%	HADS
Naheed 2021 (18)	Asia	Cross-sectional	100	41.5 ± 8.14	83	82.0%	PHQ-9
Espósito 2021 (19)	Americas	Cross-sectional	1,518	41 ± 7	1518	43.2%	HADS
Dabas 2020 (20)	Asia	Case–control	86	NR	58	12.8%	PHQ-9
Jawaid 2020 (21)	Asia	Cross-sectional	195	31.29 ± 5.34	159	10.8%	HADS
França 2020 (22)	Americas	Case–control	24	39.5 ± 7.5	24	37.5%	HADS
Shi 2019 (23)	Asia	Cross-sectional	96	40.66 ± 10.91	96	19.2%	SDS
Deshpande 2018 (24)	Asia	Case–control	50	33.4 ± 7.45	44	42.0%	HADS
Kanish 2017 (25)	Asia	Cross-sectional	123	34.42 ± 8.1	100	35.0%	PHQ-9
D’Elia 2017 (26)	Americas	Case–control	119	39.0 ± 8.2	NR	23.5%	Medical history
Fatma 2016 (27)	Africa	Cross-sectional	30	34.6	30	16.7%	HADS
Jaiswal 2016 (28)	Asia	Case–control	50	39.78 ± 7.65	40	84.0%	HDRS
Rodriguez-Arambula 2014 (29)	Americas	Cross-sectional	100	45	100	53.0%	HADS
Bashir 2010 (30)	Asia	Cross-sectional	8	NR	NR	37.5%	I.C.D-10
Zhang 2010 (31)	Asia	Cross-sectional	307	NR	298	81.1%	SDS

I.C.D-10, International Classification of Diseases Tenth Edition; HADS, Hospital Anxiety and Depression Scale; HDRS, Hamilton Depression Rating Scale; PHQ-9, Patient Health Questionnaire-9; SDS, Self-Rating Depression Scale; NR, not reported.

### 3.3 Pooled prevalence of depression

**Figure 2:**
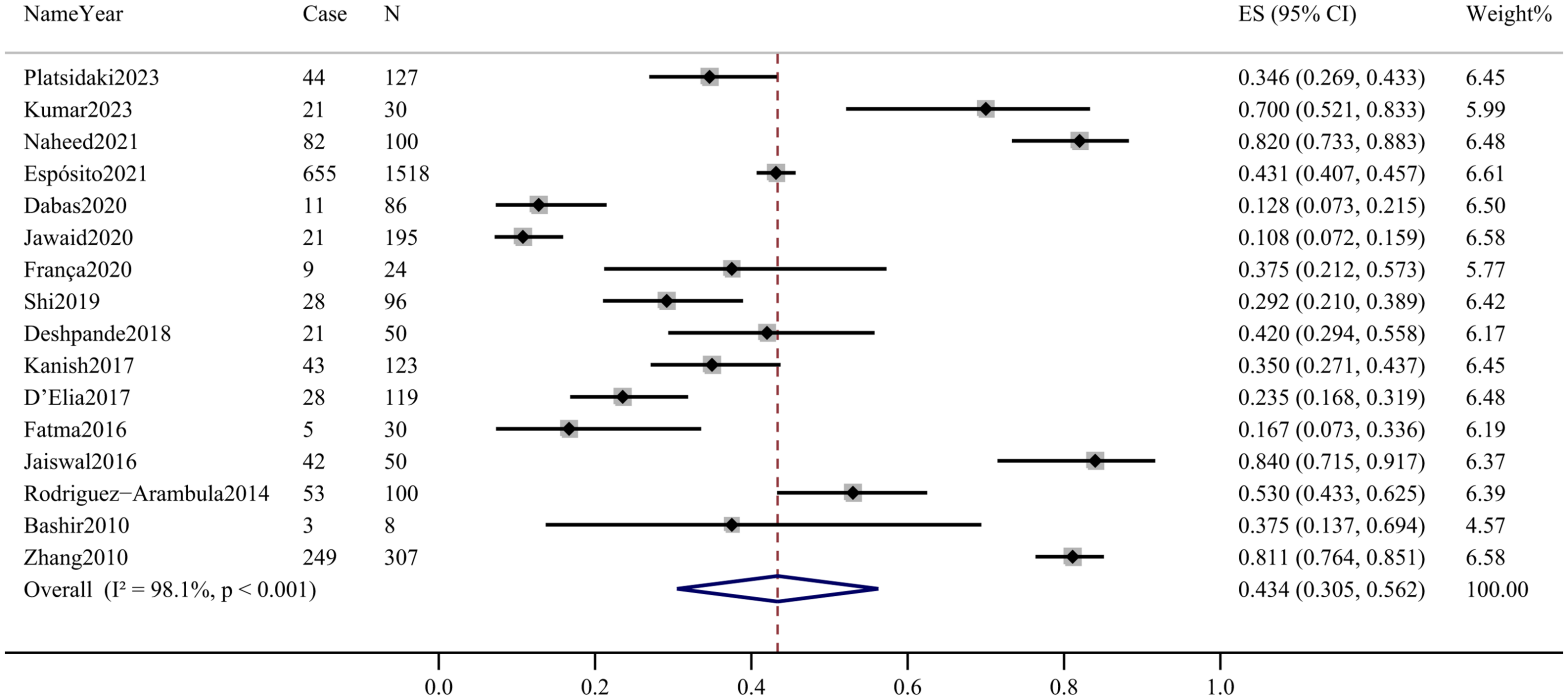
Prevalence of depression in melasma patients.

Sixteen articles assessed the prevalence of depression in patients with melasma. The prevalence ranged from 10.8% (95% CI 7.2–15.9%) to 84.0% (95% CI 71.5–91.7%). Among melasma patients, the pooled prevalence of depression was 43.4% (95% CI 30.5–56.2%, *Q*-value = 808.859, d.f. = 15, *p* < 0.001, tau^2^ = 0.065, *I^2^* = 98.1%) based on the random-effects model. There was considerable heterogeneity across the studies included in this meta-analysis. The forest plot is displayed in Figure 2.

### 3.4 Subgroup analysis

All subgroup analyses for the pooled prevalence of depression among melasma patients are summarized in Table 2. Among studies based on I.C.D-10 or medical history to identify depressive disorders, the pooled prevalence of depressive disorders among patients with melasma was 24.4% (95% CI 16.8–31.6%). While the studies assessed the prevalence of depressive symptoms among patients with melasma using validated self-reported scales, the pooled prevalence of depressive symptoms was 45.1% (95% CI 31.2–59.0%). There were significant differences between the subgroups (*p* = 0.009). With regard to geographic regions, the pooled prevalence of depression in patients with melasma in Asia was the highest at 48.5% (95% CI 26.0–71.0%). The prevalence in the Americas (North America and South America) was the next highest at 39.3% (95% CI 27.3–51.4%). The prevalence of depression was lowest in others at 26.4% (95% CI 8.9–44.0%). However, there were no significant differences between the subgroups (*p* = 0.281). In terms of study design, the pooled prevalence of depression among melasma patients was similar between the case–control studies and cross-sectional studies at 43.2% (95% CI 23.6–62.8%) and 43.5% (95% CI 25.6–61.4%), respectively, which was not significantly different (*p* = 0.984). In the subgroup analysis, heterogeneity was high (*I^2^* ranged from 0.0 to 98.8%).

**Table 2:** Subgroup analysis to estimate the prevalence of depression.

Subgroups	No. of studies	Prevalence (95%CI)	Value of *p*	*I^2^*	Value of *p* in between-group comparison
Assessment					
Depressive disorder	2	24.2% (95% CI 16.8–31.6%)	0.426	0%	0.009
Depressive symptom	14	45.1% (95% CI 31.2–59.0%)	<0.001	98.3%	
Region					
Asia	10	48.5% (95% CI 26.0–71.0%)	<0.001	98.8%	0.281
Americas	4	39.3% (95% CI 27.3–51.4%)	<0.001	89.4%	
Others	2	26.4% (95% CI 8.9–44.0%)	0.025	80.2%	
Type					
Cross-sectional	9	43.5% (95% CI 25.6–61.4%)	<0.001	98.7%	0.984
Case–control	7	43.2% (95% CI 23.6–62.8%)	<0.001	96.1%	

### 3.5 Meta-regression

Meta-regression was applied to explore potential sources of between-study heterogeneity. The prevalence of depression in melasma patients was not significantly related to the publication year (*β* = −0.0127, *p* = 0.465), the sample size (*β* = 0.0023, *p* = 0.993), and the quality of the study (*β* = 0.0019, *p* = 0.967). No sources of heterogeneity were found.

### 3.6 Sensitivity analysis and publication bias

**Figure 3:**
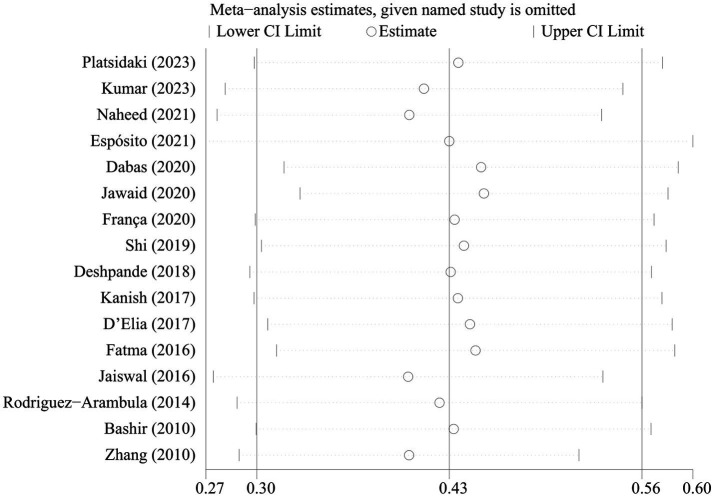
Sensitivity analysis for prevalence of depression in melasma.

The sensitivity analyses showed that the pooled prevalence of depression varied from 37.5% (95% CI 35.8–39.2%) to 48.1% (95% CI 46.4–49.8%) by excluding each study step by step (Figure 3). This indicated that no individual study had an extreme impact on the estimation of the pooled prevalence of depression in patients with melasma. Visual inspection of the funnel plot did not provide evidence of potential publication bias, as confirmed by Egger’s test (*p* = 0.999) (Supplementary Figure 1).

### 3.7 OR of depression between patients with melasma and health controls

The prevalence of depression in melasma patients was compared with healthy control groups in four studies. The pooled OR of depression among patients with melasma was 1.677 (95% CI 1.163–2.420), which meant that compared with health control groups, patients with melasma were 1.677 times more likely to show depression (Supplementary Figure 2). There was insignificant between-study heterogeneity for this outcome (*I^2^* = 0.0%, *p* = 0.606). Sensitivity analysis indicated that no individual study remarkably altered the pooled OR, suggesting that the estimated OR was reliable and stable (Supplementary Figure 3). There was no evidence of significant funnel plot asymmetry, indicating that small study effects were not present (Supplementary Figure 4).

## 4 Discussion

### 4.1 Summary of main results

This meta-analysis was, to the best of our knowledge, the first to examine the prevalence of depression in patients with melasma and assess the association between melasma and depression. The results showed that the pooled overall prevalence of depression among melasma patients was 43.4%, suggesting that approximately 4 in 10 of these patients suffered from depression. The overall pooled prevalence was approximately 12 times higher than the global estimated prevalence of depression of 3.4% (32), indicating that the existence of melasma is closely related to depression, as the pooled results showed that patients with melasma had a higher risk of depression compared to patients with the health control groups (OR = 1.677, 95% CI 1.163–2.420).

### 4.2 Subgroup analysis according to different depression assessment methods

The subgroup analysis found that the prevalence of depression varied depending on depression assessment methods. The prevalence of depressive disorders was 24.4%, which was lower than 45.1% of depressive symptoms. There was a significant difference (*p* = 0.009). This finding was consistent with the results of most studies, which indicated that the prevalence of depressive symptoms using self-report scales was significantly higher than the prevalence of depressive disorders. Perhaps it was because self-report scales prioritized sensitivity over specificity (33), and the optimal cutoff value for self-report scales among melasma patients has not been determined, with many studies using multiple cutoff points, and information on the qualifications and training of researchers conducting surveys was rarely provided, which might also affect the estimation of prevalence. The standardized clinical interview was the most widely used approach by psychiatrists to confirm the formal diagnosis of depressive disorders (34). Therefore, it was recommended to use diagnostic interviews for more large-scale research as it was effective, used strictly defined criteria for depressive disorders, and constituted the main body of clinical research on depressive disorders (34); however, these usually required a considerable amount of time and effort and were expensive. Although the self-report measure alone could not be used to determine formal diagnosis, which tended to report higher rates than diagnostic interviews, it can be used for preliminary screening or early detection of depression because it was less limited, more feasible, easier to fill out, and cheaper to use in busy clinical environments (35). Therefore, future research should develop and use more appropriate assessment tools and cutoff values in melasma, and attempt to screen for depression as much as possible in clinical practice.

### 4.3 Subgroup analysis according to different regional groups

When the pooled prevalence of depression in melasma patients was stratified across geographic regions, the highest prevalence was found in Asia at 48.5%, followed by the Americas at 39.3% and others at 26.4%. First, the reason for the difference might be related to the prevalence of melasma in different regions. Research has shown that melasma was more common in Fitzpatrick skin type IV and IV skin (5, 36), which were the main skin types in Asians. In addition, existing research indicated that some populations, including South Asians and East Asians, were more vulnerable to melasma than others, such as Europeans, Australians, and Africans (37). Second, it might be related to cultural impact. A preference for facial skin color has been shown to be prevalent in diverse cultures. In Asian culture, being fair-skinned was important (38). Furthermore, patients with melasma were mainly female, and women were more concerned about their facial appearance (39). Eventually, it might be related to the economic level. Research in the Asian region mainly focused on India and Pakistan, both of which were developing countries with relatively low *per capita* CDP (40). Economy, education, and health-related behaviors were closely related (41). Therefore, patients might lack correct disease awareness and prevention information, might not be aware of sun protection measures, and mistakenly believe that melasma was caused by undiagnosed liver or kidney problems, exacerbating psychological burden, but there were no statistically significant differences between the groups (*p* = 0.281). Our meta-analysis included very limited studies from Africa and Europe and no study from Oceania. Therefore, original studies need to be carried out in more regions in future to further investigate the depression of melasma patients.

### 4.4 Limitations

Admittedly, this study has several limitations. First, the included studies were cross-sectional or case–control studies, which were vulnerable to recall bias, selection bias, and residual confounding. The results showed that depression was closely related to melasma. Compared with the healthy control groups, patients with melasma had a higher risk of depression. However, the causal relationship between depression and melasma could not be determined. Therefore, future prospective cohort studies with large sample sizes and long follow-up periods should be conducted to explore the causal or bidirectional associations between depression and melasma. Second, most studies used self-report scales to assess the prevalence of depression, which inevitably led to reporting and recall bias. This might have affected the prevalence of depression in patients with melasma to varying degrees. No subgroup analysis was conducted on the self-reported scales as only one eligible study used the HDRS. Third, some factors have not been reported in sufficient detail, such as the severity and duration of melasma, disease stage, treatment strategy, evaluation time node, age of melasma onset, gender, education, and income. These factors could not be extracted to be usefully assessed. Future research should take into account the impact of these factors that may affect the prevalence of depression. Fourth, there were very few studies from Europe and Africa, while no study was identified from Oceania, which limited global adaptability. Therefore, caution should be exercised about the actual prevalence. Finally, there was a high level of heterogeneity across studies included in our analysis, as reported previously in the meta-analyses of prevalence data (42). This showed that the variability in depression prevalence measurements was due to the heterogeneity between the studies rather than chance.

## 5 Conclusion

The prevalence of depression in patients with melasma is high, and there is a remarkable relevance between melasma and depression. In future clinical practice, more attention should be paid to the screening of depression in melasma patients, and the mutual negative impact of depression and melasma should be realized; in the treatment of melasma, we should pay sufficient attention to the identification, prevention, and psychological management of depression. These will improve the quality of life, improve therapeutic outcomes, and reduce the social, medical, and economic burden of the disease. In addition, it is suggested that a large-scale prospective cohort study will be conducted in future to assess the prevalence of depression in melasma patients and the causal relationship between depression and melasma. Apart from investigating the effect of melasma on depression, the impact of depression on melasma should also be studied.

## Author contributions

WC: Writing – original draft. YW: Writing – original draft. YS: Writing – original draft. CG: Writing – original draft. JL: Writing – original draft.
