# Supplementary material for: Prevalence of depression in melasma: a systematic review and meta-analysis
Source: Front Psychiatry. 2024 Jan 8;14:1276906. doi: 10.3389/fpsyt.2023.1276906 (PMC10800906; doi:10.3389/fpsyt.2023.1276906)
Supplement: Supplementary file 1 [file Data_Sheet_1.PDF]

Supplementary Material

Prevalence of Depression in Melasma: A Systematic Review and Meta-analysis

Wenjing Chen, Yue Wan, Yuan Sun, Changyong Gao, Jianhong Li[*]

*** Correspondence:** Jianhong Li: gracelee100@bucm.edu.cn

1 Search strategy

(((((((((((("Depression"[Mesh] OR "Depressive Disorder"[Mesh] OR "Depressive Disorder, Major"[Mesh] OR "Depressive Disorder, Treatment-Resistant"[Mesh] OR "Depression, Postpartum"[Mesh] OR "Bipolar Disorder"[Mesh] OR "Adjustment Disorders"[Mesh] OR "Affective Disorders, Psychotic"[Mesh] OR "Dysthymic Disorder"[Mesh]) OR ("Mood Disorders"[Mesh])) OR ("Antidepressive Agents"[Mesh])) OR ("Mental Health"[Mesh])) OR ("Psychological Distress"[Mesh])) OR ("Mental Disorders"[Mesh])) OR ("Stress, Psychological"[Mesh])) OR ("Burnout, Psychological"[Mesh])) OR (((Depressive Symptoms[Title/Abstract]) OR (Depressive Symptom[Title/Abstract])) OR (Symptom, Depressive[Title/Abstract])) OR (Emotional Depression[Title/Abstract])) OR (Depression, Emotional[Title/Abstract])) OR (Depressive Disorders[Title/Abstract])) OR (Disorder, Depressive[Title/Abstract])) OR (Disorders, Depressive[Title/Abstract])) OR (Neurosis, Depressive[Title/Abstract])) OR (Depressive Neuroses[Title/Abstract])) OR (Depressive Neurosis[Title/Abstract])) OR (Neuroses, Depressive[Title/Abstract])) OR (Depression, Endogenous[Title/Abstract])) OR (Depressions, Endogenous[Title/Abstract])) OR (Endogenous Depression[Title/Abstract])) OR (Endogenous Depressions[Title/Abstract])) OR (Depressive Syndrome[Title/Abstract])) OR (Depressive Syndromes[Title/Abstract])) OR (Syndrome, Depressive[Title/Abstract])) OR (Syndromes, Depressive[Title/Abstract])) OR (Depression, Neurotic[Title/Abstract])) OR (Depressions, Neurotic[Title/Abstract])) OR (Neurotic Depression[Title/Abstract])) OR (Neurotic Depressions[Title/Abstract])) OR (Melancholia[Title/Abstract])) OR (Melancholias[Title/Abstract])) OR (Unipolar Depression[Title/Abstract])) OR (Depression, Unipolar[Title/Abstract])) OR (Depressions, Unipolar[Title/Abstract])) OR (Unipolar Depressions[Title/Abstract])) OR (Depressive Disorders, Major[Title/Abstract])) OR (Major Depressive Disorders[Title/Abstract])) OR (Major Depressive Disorder[Title/Abstract])) OR (Paraphrenia, Involutional[Title/Abstract])) OR (Involutional Paraphrenia[Title/Abstract])) OR (Involutional Paraphrenias[Title/Abstract])) OR (Paraphrenias, Involutional[Title/Abstract])) OR (Psychosis, Involutional[Title/Abstract])) OR (Involutional Psychoses[Title/Abstract])) OR (Involutional Psychosis[Title/Abstract])) OR (Psychoses, Involutional[Title/Abstract])) OR (Depression, Involutional[Title/Abstract])) OR (Involutional Depression[Title/Abstract])) OR (Melancholia, Involutional[Title/Abstract])) OR (Involutional Melancholia[Title/Abstract]))))) OR ((("Anxiety"[Mesh] OR "Performance Anxiety"[Mesh] OR "Anxiety Disorders"[Mesh] OR "Anti-Anxiety Agents"[Mesh] OR "Anxiety, Separation"[Mesh] OR "Phobia, Social"[Mesh]) OR ("Neurotic Disorders"[Mesh])) OR ((((((((((((((((((((((Angst[Title/Abstract]) OR (Social Anxiety[Title/Abstract])) OR (Anxieties,

Social[Title/Abstract])) OR (Anxiety, Social[Title/Abstract])) OR (Social Anxieties[Title/Abstract])) OR (Hypervigilance[Title/Abstract])) OR (Nervousness[Title/Abstract])) OR (Anxiousness[Title/Abstract])) OR (Anxieties, Performance[Title/Abstract])) OR (Anxiety, Performance[Title/Abstract])) OR (Performance Anxieties[Title/Abstract])) OR (Anxiety Disorder[Title/Abstract])) OR (Disorder, Anxiety[Title/Abstract])) OR (Disorders, Anxiety[Title/Abstract])) OR (Neuroses, Anxiety[Title/Abstract])) OR (Anxiety Neuroses[Title/Abstract])) OR (Anxiety States, Neurotic[Title/Abstract])) OR (Anxiety State, Neurotic[Title/Abstract])) OR (Neurotic Anxiety State[Title/Abstract])) OR (Neurotic Anxiety States[Title/Abstract])) OR (State, Neurotic Anxiety[Title/Abstract])) OR (States, Neurotic Anxiety[Title/Abstract])))) OR ((((((((((((((((Depression[Title/Abstract]) OR (Depressive Disorder[Title/Abstract])) OR (Depressive Disorder, Major[Title/Abstract])) OR (Depressive Disorder, Treatment-Resistant[Title/Abstract])) OR (Depression, Postpartum[Title/Abstract])) OR (Bipolar Disorder[Title/Abstract])) OR (Adjustment Disorders[Title/Abstract])) OR (Affective Disorders, Psychotic[Title/Abstract])) OR (Dysthymic Disorder[Title/Abstract])) OR (Antidepressive Agents[Title/Abstract])) OR (Mood Disorders[Title/Abstract])) OR (Mental Health[Title/Abstract])) OR (Psychological Distress[Title/Abstract])) OR (Mental Disorders[Title/Abstract])) OR (Stress, Psychological[Title/Abstract])) OR (Burnout, Psychological[Title/Abstract]))) OR (((((((Anxiety[Title/Abstract]) OR (Performance Anxiety[Title/Abstract])) OR (Anxiety Disorders[Title/Abstract])) OR (Anti-Anxiety Agents[Title/Abstract])) OR (Anxiety, Separation[Title/Abstract])) OR (Phobia, Social[Title/Abstract])) OR (Neurotic Disorders[Title/Abstract]))) AND (("Melanosis"[Mesh]) OR ((((((((Melanoses[Title/Abstract]) OR (Melanism[Title/Abstract])) OR (Freckles[Title/Abstract])) OR (Freckle[Title/Abstract])) OR (Chloasma[Title/Abstract])) OR (Chloasmas[Title/Abstract])) OR (Melasma[Title/Abstract])) OR (Melasmas[Title/Abstract])))

2 Supplementary Figures and Tables

2.1 Supplementary Figures

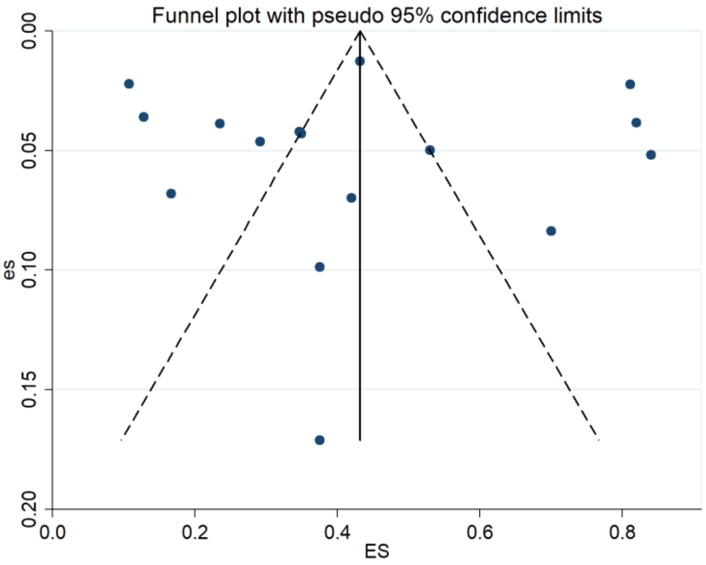

Figure 1 Funnel plot for the prevalence of depression in melasma.

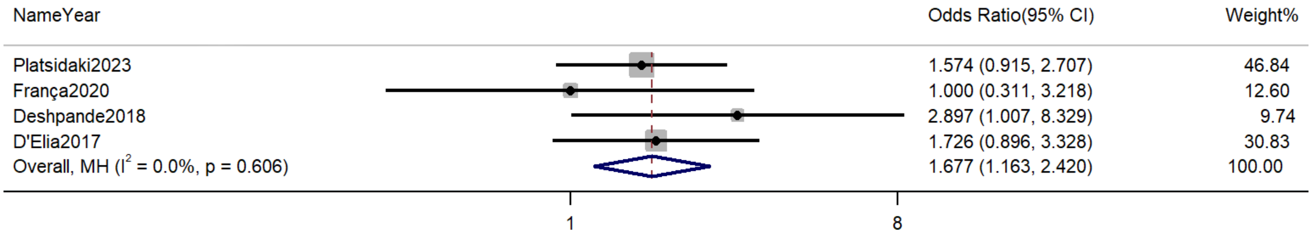

Figure 2 OR of depression between patients with melasma and health controls.

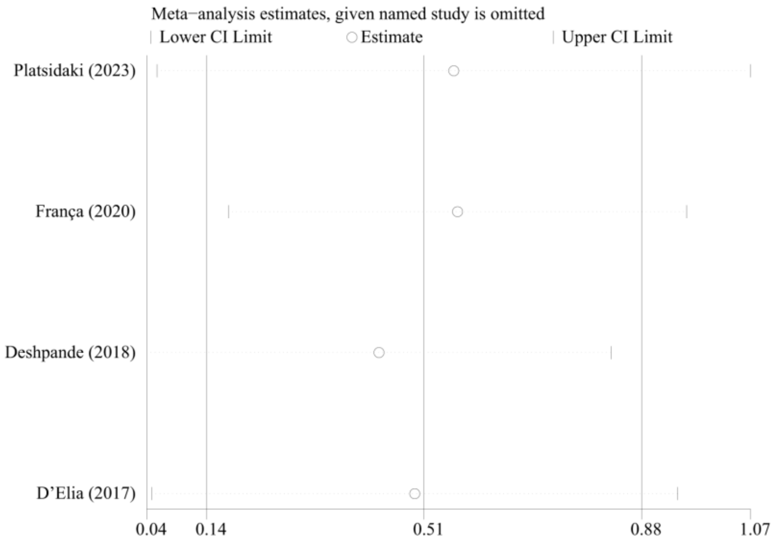

Figure 3 Sensitivity analysis for the OR.

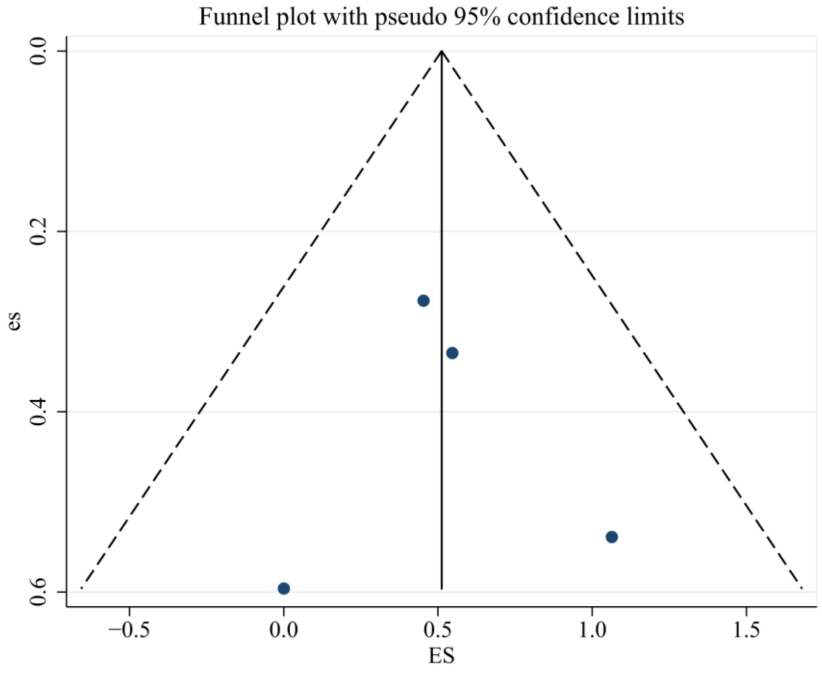

Figure 4 Funnel plot for OR.

2.2 Supplementary Table

Table 1 Quality assessment of included studies.

Author, year	Q1	Q2	Q3	Q4	Q5	Q6	Q7	Q8	Q9	Total[a]	Quality[b]
Platsidaki 2023	Y	U	N	Y	Y	Y	U	Y	Y	6	High
Kumar 2023	Y	U	N	Y	U	Y	U	N	U	3	Low
Naheed 2021	Y	U	N	Y	Y	Y	U	Y	Y	6	High
Espósito 2021	U	U	Y	Y	U	Y	U	Y	U	4	Median
Dabas 2020	Y	U	N	N	Y	Y	U	Y	U	4	Median
Jawaid 2020	Y	N	N	Y	Y	Y	U	Y	Y	6	High
França 2020	Y	U	N	Y	Y	Y	U	Y	Y	6	High
Shi 2019	N	U	N	Y	Y	Y	Y	Y	Y	6	High
Deshpande 2018	Y	U	N	Y	Y	Y	Y	Y	Y	7	High
Kanish 2017	Y	U	N	Y	U	Y	Y	Y	U	5	Median
D'Elia 2017	Y	N	N	Y	Y	U	U	Y	Y	5	Median
Fatma 2016	U	U	N	Y	Y	Y	U	U	Y	4	Median
Jaiswal 2016	Y	N	N	Y	Y	Y	U	Y	Y	6	High
Rodriguez-Arambula 2014	U	U	N	Y	U	Y	U	U	U	2	Low
Bashir 2010	N	U	N	N	U	Y	Y	N	U	2	Low
Zhang 2010	Y	U	N	Y	Y	Y	U	U	Y	5	Median

Note. Y, yes; N, no; U, unclear; Q, question; [a] Quality score based on the Joanna Briggs Institute (JBI) Critical Joanna Briggs Institute's critical appraisal checklist for studies reporting prevalence data; Scoring 1 = yes, 0 = no/unclear; [b] Classification as follow: High-quality if total score > 5, medium-quality if total score 4-5, low-quality if total score< 4.

Q1	Was the sample frame appropriate to address the target population?
Q2	Were study participants sampled in an appropriate way?
Q3	Was the sample size adequate?
Q4	Were the study subjects and the setting described in detail?
Q5	Was the data analysis conducted with sufficient coverage of the identified sample?
Q6	Were valid methods used for the identification of the condition?
Q7	Was the condition measured in a standard, reliable way for all participants?
Q8	Was there appropriate statistical analysis?
Q9	Was the response rate adequate, and if not, was the low response rate managed appropriately?
